# Investigation of the Impact of Wellinks on the Quality of Life and Clinical Outcomes in Patients With Chronic Obstructive Pulmonary Disease: Interventional Research Study

**DOI:** 10.2196/47555

**Published:** 2024-02-09

**Authors:** Kerri A Pierz, Nicholas Locantore, Gretchen McCreary, Robert J Calvey, Nickole Hackney, Pooja Doshi, John Linnell, Abirammy Sundaramoorthy, Carol R Reed, Julie Yates

**Affiliations:** 1 Wellinks (Convexity Scientific, Inc) New Haven, CT United States; 2 COPD Foundation Miami, FL United States

**Keywords:** chronic obstructive pulmonary disease, COPD, health coaching, pulmonary rehabilitation, remote care, disease management, patient engagement, Wellinks, digital health, adult, mobile app, remote model of care, mobile phone

## Abstract

**Background:**

Wellinks is a remote disease management solution that provides novel chronic obstructive pulmonary disease (COPD) care delivery.

**Objective:**

This study evaluated the satisfaction, engagement, and clinical outcomes of Wellinks participants. This study also investigated the cadence of health coaching for patients with COPD.

**Methods:**

A 24-week interventional study was conducted by Wellinks and the COPD Foundation in 2022. Adults with COPD were recruited by the COPD Foundation in the United States and determined to be eligible if they had phone and internet access, owned a smartphone, and were not currently participating in pulmonary rehabilitation. All study participants provided written informed consent. The Wellinks solution included remote health coaching, pulmonary rehabilitation, and group education; participants were provided the Wellinks app and smart spirometry and pulse oximetry devices. Participants were offered 6 coaching sessions in the first 12 weeks. For the second 12-week period, participants either reduced frequency or discontinued coaching; all other components of the Wellinks solution remained unchanged. The COPD Self-Efficacy Scale, Modified Medical Research Council dyspnea scale, pulmonary function, pulse oximetry, and patient-reported healthcare resource utilization were the clinical outcome measures. Nonclinical outcomes included engagement and satisfaction with Wellinks and net promoter score.

**Results:**

In total, 141 adults consented and completed Wellinks onboarding; 84.4% (n=119) of whom remained engaged throughout the 24-week study. Participants had a mean age of 70 (SD 7.8; range 48-88) years, and 55.7% (n=78) were female. Most participants (n=119, 84.4%) completed all 6 coaching sessions during the first 12-week period. Compliance with spirometer and pulse oximeter use was 82.3% and 89.4%, respectively, at week 1 but waned over the study period to 8.5% and 9.2%, respectively, at the end of the study. Participants indicated a high degree of satisfaction with Wellinks, with 95.5% (n=85) and 91% (n=81) of participants indicating that they agreed or strongly agreed that the educational content and health coaching, respectively, were valuable. At the end of the study, the net promoter score was +64 and +55 in the coaching continuation and discontinuation arms, respectively. A significant improvement from baseline to end of the study was observed in the COPD Self-Efficacy Scale total score (*P*<.001) and domain scores (*P*<.001 for each domain). In total, 35.1% (n=27) of participants improved by at least 1 category of change on the 5-point Modified Medical Research Council dyspnea scale from baseline to week 24.

**Conclusions:**

This study confirmed the feasibility of using a remote model of care delivery to support people living with COPD. The insights gained in this study have allowed for further refinement and personalization of the Wellinks care model. Findings related to the combined use of technology and personal care delivery should be considered by others developing remote disease management tools.

**Trial Registration:**

ClinicalTrials.gov NCT05259280; https://clinicaltrials.gov/ct2/show/NCT05259280

## Introduction

Chronic medical conditions are highly prevalent among US adults and require long-term management strategies that invoke the need for participatory medicine. Strategies and services that support patient self-management are capable of reducing the impact of chronic disease on the individual and on the health care system [1]. Self-management strategies should optimize and preserve health, reduce symptoms and the impact of disease on daily life, improve quality of life, and build patient and provider relationships [1].

Chronic disease management programs have evolved over time to deliver remote health care and are generally inclusive of both technological (eg, wearables and mobile apps) and personal components, such as coaching or counseling [2]. While society is immersed in the Internet of Things, remote health care delivery needs to be more than biometric monitoring alone and to be effectively integrated with the delivery of care [3]. Although available devices can track mobility, heart rate, oxygen levels, blood pressure, cardiac activity, and body temperature and detect posture and falls (and more), the use of the data is limited if it is not integrated, shared, and applied to the delivery of care. Peyroteo et al [3] cite more than 100,000 apps that have been created to use data from various biometric sensors but also note the lack of integration with care systems as a key limitation to maximizing health outcomes.

Chronic obstructive pulmonary disease (COPD) is a chronic medical condition of the lungs affecting more than 16 million adults in the United States [4]. It is among the top causes of disability worldwide and is projected to become the leading cause of death by disease by 2030 [5]. Annual US health care expenditures on COPD exceed US $49 billion, with employer, federal, and state spending on health care services reaching unsustainable levels [6]. Costs aside, the toll on those struggling with respiratory diseases has been widely reported to lead to the significant presence of comorbidities such as cardiac diseases, diabetes, hypertension, osteoporosis, and mental health disorders [7].

Wellinks goes beyond remote patient monitoring. Wellinks is a COPD disease management solution that pairs technology with personalized health coaching and respiratory therapy services to offer a novel approach to COPD remote disease management. Wellinks is a care partner delivering remotely accessible pulmonary rehabilitation, clinical coaching, a mobile app, and connected devices for home monitoring of pulmonary function.

In a previously published 8-week pilot study of Wellinks, it was demonstrated that patients with COPD with an average age of 79.6 years were able to successfully use the devices provided (ie, Flyp nebulizer [Convexity Scientific, Inc], Smart One spirometer [Medical International Research], and NoninConnect smart pulse oximeter 3230 [Nonin Medical, Inc]) as well as enter data into the Wellinks mobile app [8]. Study participants reported the app to be valuable (13/16, 81%) and easy to use (15/16, 94%). This feasibility study provided preliminary evidence for the willingness and capability of this patient population to successfully use the digital tools provided by Wellinks [8].

Since the original pilot study, the Wellinks solution has expanded to include respiratory therapy and health coaching services, in addition to some modifications of the technological components described earlier. With such iteration, not only was it important to replicate the previously reported feasibility results but also to explore clinical outcome measures and refine the duration and frequency of health coaching. Described herein is the ASPIRE study conducted in partnership with the COPD Foundation that was designed to explore clinical and nonclinical outcomes associated with the use of the updated Wellinks solution inclusive of both personal (health coaching and respiratory therapy) and technological components. The objectives of this study were to determine to what degree study participants would engage with the various components of the Wellinks solution over time and whether any clinical outcomes could be identified to correlate with engagement. This study also sought to collect qualitative feedback on the components of the Wellinks solution and observe any impact of decreased frequency of engagement with the Wellinks team, in service of refining the care delivery model offered by Wellinks.

## Methods

### Study Design

This 24-week, prospective, interventional research study of the Wellinks COPD solution including the use of Bluetooth-connected devices, patient mobile app, COPD-related education, and health coaching services was conducted from December 2021 through September 2022. This study was designed to gather data on the quality of life and clinical impact of the use of the Wellinks COPD solution, in addition to collecting feedback from patients and investigators to inform further optimization of this intervention. The study was posted to ClinicalTrials.gov (NCT05259280).

### Ethical Considerations

The conduct and performance of this study were in accordance with applicable sponsor and investigator responsibilities as described in Title 21 Code of Federal Regulations 812 and other Good Clinical Practice guidance. Institutional review board (IRB) or ethics committee oversight was required as human participants or data from humans were used. IRB approval of the study protocol and study-related materials was obtained from Western IRB prior to beginning any study-related procedures (IRB protocol 20141136).

### Recruitment

Eligible participants were recruited through the COPD Foundation Patient-Powered Research Network, COPD360Social, and various newsletters. Eligible patients were invited to participate in the study. Participants were required to give informed consent before study-specific procedures could proceed. Eligible study participants included adults (≥18 years of age) with a diagnosis of COPD. Participants had to have access to a home telephone (landline or mobile) and the internet and must have had a smartphone (ie, iPhone 6S or later model, running iOS 14.0 or later model, and Android 6 or later model). Individuals must have been proficient in the English language, living or staying in the United States throughout the study duration, willing and able to comply with study requirements, and able to provide written informed consent. Exclusion criteria included current participation in other interventional clinical trials and current participation in a pulmonary rehabilitation program.

### Intervention

The Wellinks COPD solution combined personal and technological elements to remotely support enrolled participants living with COPD. The personal elements of the program included one-on-one access to health and wellness coaches or nurse practitioners trained in health coaching methodologies. Health coaches provided support to participants via phone, video, and text interactions throughout the study period. The role of the coach was to support the participant by providing disease-state and treatment-related education, establishing and supporting the attainment of individual health goals, and encouraging adherence to treatment and attendance at clinic visits. Remote pulmonary rehabilitation programs were provided by the health coach, personalized for each participant, and included individual home-based exercise guides or videos and group educational sessions led by Wellinks respiratory therapists that were held remotely.

The technological elements of the program included a mobile app and Bluetooth-enabled medical devices. The Wellinks mobile app downloaded to an iOS (iPhone) or Android device allowed participants to connect with their coach; track goals, medications, and symptoms; and review data from the connected devices provided. The Smart One personal spirometer was used by participants at home to collect peak expiratory flow (PEF) and forced expiratory volume in 1 second (FEV_1_). The NoninConnect 3230 pulse oximeter was used at home to measure blood oxygenation (saturation of peripheral oxygen [SpO_2_]) and pulse rate. Data from both devices were transmitted via Bluetooth to the participant’s smartphone. Technical support was available to all study participants throughout the duration of the study to answer questions about the technological components or to troubleshoot any issues.

### Study Procedures

All baseline assessments were collected via a survey of all consented participants followed by an onboarding call between a Wellinks coach and the participant to ensure the technical set-up of the app and devices and introduce the coaching process. Participants were instructed to use the connected devices (pulse oximeter and spirometer) at least once a week throughout the duration of the study, use the app to track symptoms and medications, and monitor their own spirometry and pulse oximetry data throughout the study. The frequency of use of each component of Wellinks was recommended to each participant, but in order to best emulate real-world use, the health coaches encouraged but did not mandate the use of all available components.

Participants were sequentially assigned at the time of enrollment in an alternating fashion to arm 1 or arm 2 by the Wellinks head health coach. Participants were not informed of this assignment until the completion of the first 12-week period. In the first 12-week study period, health coaching was offered to all participants in the form of one-on-one 30-minute remote sessions scheduled every other week for a total of 6 sessions over the 12-week period. In addition, participants were instructed to individually perform the remote pulmonary rehabilitation program as directed by their health coach, and they were invited to attend group educational sessions held weekly throughout the study period. In the second 12-week study period, all components of the program remained the same except for the level of personal contact with health coaches. Participants assigned to arm 1 continued with a lower level of engagement with their coach in the form of SMS text messaging or up to 3 brief check-in meetings (15-minute sessions) for an additional 12 weeks. Participants who were assigned to arm 2 discontinued access to the Wellinks health coaches for the second 12 weeks of the study.

### Outcomes

The nonclinical objectives of this study were to describe the experience of patients using the Wellinks solution through the assessment of patient engagement as well as by patient-reported satisfaction. Outcome measures included compliance with protocol-recommended device use, compliance with attendance at scheduled coaching sessions, ratings of the degree to which participants valued individual components of the Wellinks COPD solution, and net promoter score (NPS; ie, “How likely is it that you would recommend Wellinks to a friend or colleague?” 0=not at all likely to 10=extremely likely).

Spirometer and pulse oximeter data could be synced with the Wellinks app; as such, the use of data from the app provided the necessary data to determine whether participants used these at-home devices. However, the spirometer results could only be viewed by the participants via the app, while the pulse oximeter could be viewed independently of the app. Therefore, the compliance with the pulse oximeter uniquely may be underestimated.

The clinical objectives of this study were to determine whether the use of the Wellinks COPD solution could improve the quality of life for patients with COPD, reduce healthcare resource utilization (HRU) over time, and improve pulmonary function as measured by connected devices. Quality of life was indirectly ascertained by the interpretation of results from the COPD Self-Efficacy Scale (CSES) and Modified Medical Research Council (mMRC) dyspnea scale, based on known correlations reported in the published literature [9]. Pulmonary function was measured using at-home devices to collect FEV_1_, PEF, and SpO_2_. Patient-reported HRU was collected via survey.

The CSES is used to assess the confidence of a patient related to their ability to avoid breathing difficulty based on responses to 34 questions within 5 domains; each question is scored from 1 (not at all confident) to 5 (very confident) [10]. A higher score thus reflects a greater degree of confidence on the part of the respondent. Total scores can range from 34 to 170. The CSES is divided into 5 domains: negative affect, intense emotional arousal, physical exertion, weather or environmental factors, and behavioral risk factors [10].

The mMRC dyspnea scale provides an assessment of a patient’s shortness of breath and its impact on daily activities. At onboarding, data from the mMRC dyspnea scale were combined with patient-reported exercise habits to individualize the remote pulmonary rehabilitation program to be suitable to each study participant’s level of functioning. Participants were assigned to 1 of 6 different exercise programs based on mMRC score (low=0, 1, or 2 or high=3 or 4) and self-reported level of exercise (low, medium, or high) at baseline. mMRC was also assessed at week 12 and week 24 to explore changes over time.

Pulmonary function was measured as a change from baseline to week 12 and week 24 in FEV_1_, PEF, and SpO_2_ based on patient use of the Bluetooth-connected spirometer and pulse oximeter provided. When used and connected, these data were captured in the Wellinks app. At the start of the study, participants were asked to use the pulse oximeter and spirometer at least weekly throughout the duration of the study.

Patient-reported HRU was collected through a web-based survey and relied upon individual recall. HRU is a reflection of the patient’s desire or need to seek care and is a measure that can be used to inform the economic impact of an intervention. At baseline, participants were asked to report certain HRU (ie, COPD-related physician visits, emergency department visits, and hospital admissions) in the 3-month and 1-year periods prior to enrollment. Participants were asked the same HRU questions at week 12 and week 24 of the study, each with a 3-month recall period. Outcome measures were assessed at baseline, week 12, and week 24 of the study. Any adverse events or serious adverse events were collected via spontaneous reporting from the study participants.

### Statistical Analyses

The planned sample size for this study (n=150) was based on the expected feasibility for recruitment. No formal statistical power calculations were performed to size this study.

Study data were summarized for arm 1, arm 2, and full study cohort. Unless otherwise specified, data were summarized as number and percentage for categorical variables and as mean and SD for continuous variables. All statistical analyses were exploratory in nature. *P* values for statistical tests are 2-sided tests and not adjusted for multiplicity. Analyses of change from baseline values at week 12 and week 24 were performed for each arm and the full study cohort using 2-tailed *t* tests. Least squares (LS) mean and LS mean change from baseline at each time point with corresponding SEs for change and *P* values were produced.

For mMRC, a responder was defined as a participant with an improvement from baseline of 1 category or more. For example, a participant who changes from “3: I have to stop for breath after walking for ~100 yards” at baseline to a postbaseline value of “2: I walk slower than others of my age because I am out of breath, or I have to stop often to catch my breath” would be classified as a responder at that time point. Participants who either remain in the same category or worsen were classified as nonresponders.

## Results

### Demographics and Baseline Characteristics

**Figure 1:**
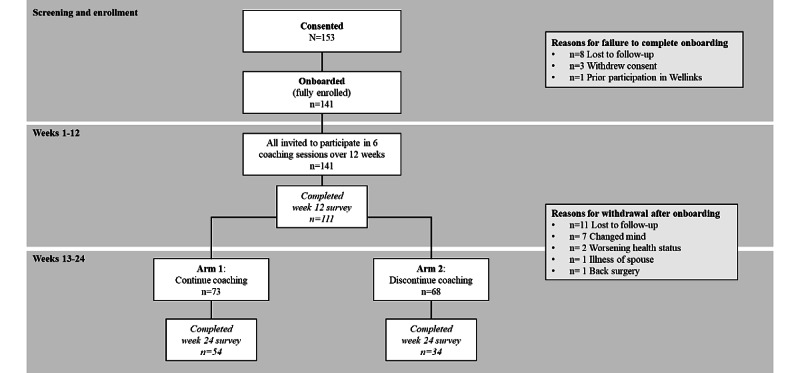
Study flow diagram. Flow of participants through the study protocol is described as inclusive of the number of individuals consented to participate (N=153) and enrolled (n=141), followed by completion of certain milestones throughout the 24-week study period. Reasons for withdrawals from the study are reported.

A total of 153 individuals were consented in this study, of whom 141 were fully enrolled (ie, consented and completed onboarding of devices and app). Disposition of participants in the study is described in Figure 1.

The demographics of the study population are presented in Table 1. Study participants had a mean age of 70 (SD 7.8; range 48-88) years, 78% (n=110) were 65 years of age or older, and 55.3% (n=78) were female. The population was 90.8% (n=128) White and 97.9% (n=138) non-Hispanic or Latino. There were no statistically significant differences between the 2 treatment arms for any of the demographic variables.

In the study population, 83.7% (n=118) of participants were former smokers (with 77.1% [n=91] of these having quit more than 5 years ago), and 9.2% (n=13) were current smokers with a mean use of 12 (SD 6.1) cigarettes per day at the time of the study.

It was self-reported that 82.3% (n=116) of the population was under the care of a pulmonologist for their COPD, and 39.7% (n=56) reported a primary care physician participating in the management of their COPD alone or together with the pulmonologist. A majority (74.5%, n=105) of participants had been living with COPD for at least 5 years at the time they were enrolled in this study. The severity of disease was self-reported to be moderate (51.1%, n=72) or severe (39.7%, n=56), and 58.2% (n=82) lacked an exercise plan at the study start. Some degree of home oxygen use was reported by 61% (n=86) of study participants (45.4% [n=64] daily use and 15.6% [n=22] as-needed use).

More than half of the study population self-reported emphysema (71.6%, n=101) or bronchitis (53.9%, n=76). High blood pressure (56.7%, n=80) and anxiety (46.1%, n=65) were among the most common nonrespiratory medical conditions reported by the study population.

**Table 1:** Summary of participant demographics. Descriptive information about the study cohort at baseline is presented for the combined cohort and for individuals assigned to arm 1 and arm 2 separately.

Parameter and statistic or variable		Combined (n=141)	Arm 1 (n=73)	Arm 2 (n=68)
**Age (years)**				
	Mean (SD)	70 (7.6)	70 (7.8)	70 (7.5)
	Range	48-88	48-87	49-88
	65 years or older, n (%)	110 (78)	60 (82.2)	50 (73.5)
Sex (female), n (%)		78 (55.3)	38 (52.1)	40 (58.8)
**Race, n (%)**				
	Black	5 (3.5)	3 (4.1)	2 (2.9)
	White	128 (90.8)	65 (89)	63 (92.6)
	Others	3 (2.1)	1 (1.4)	2 (2.9)
	Unknown or declined	5 (3.5)	4 (5.5)	1 (1.5)
**Ethnicity, n (%)**				
	Hispanic or Latino	1 (0.7)	0 (0)	1 (1.5)
	Non-Hispanic or Latino	138 (97.9)	73 (100)	65 (95.6)
	Unknown or declined	2 (1.4)	0 (0)	2 (2.9)
Height (inches), mean (SD)		67 (4.1)	67 (4.0)	66 (4.2)
Weight (lb), mean (SD)		180 (46.9)	184 (46.5)	175 (47.1)
**Smoking status at baseline, n (%)**				
	Current smoker	13 (9.2)	8 (10.6)	5 (7.4)
	Former smoker	118 (83.7)	58 (79.5)	60 (88.2)
	Never smoked	10 (7.1)	7 (9.6)	3 (4.4)

### Nonclinical Outcomes

#### Engagement Metrics

In total, 84.4% (n=119) of all participants completed all 6 coaching sessions in the first 12-week period of the study. Among participants assigned to arm 1 (continued coaching), attendance diminished session-to-session in the second 12-week period of the study, with only 52.1% (n=38) of those assigned to arm 1 completing the third (final) 15-minute coaching session in the second study period.

Participants were advised to use the Bluetooth-connected spirometer and pulse oximeter at least weekly throughout the 24-week duration of the study. Spirometer compliance peaked at the start of the study with 82.3% (n=116) of participants compliant during week 1, but compliance decreased to a smaller proportion of participants at week 12 (n=59, 41.8%) and week 24 (n=12, 8.5%). Similarly, pulse oximeter compliance also peaked at week 1 with 89.4% (n=126) of participants using the pulse oximeter as recommended, and this rate of compliance decreased to 42.6% (n=60) and 9.2% (n=13) at week 12 and week 24, respectively. Compliance with the spirometer or the pulse oximeter use did not differ by treatment arm. For the entire study period, 21.3% (n=30) and 22.6% (n=32) of participants were compliant with spirometer and pulse oximeter use, respectively, for more than 75% of the study period (18 or more of 24 weeks), while 30.5% (n=43) and 29.1% (n=41) were compliant with spirometer and pulse oximeter use, respectively, for 25% or less of the study period (6 or less weeks).

Similar rates of compliance were observed with the use of the Wellinks app; compliance with mobile app use peaked at the start of the study with 94.3% (n=133) compliance in week 1, which declined to 50.4% (n=71) and 22.7% (n=32) at week 12 and week 24, respectively. For the entire study period, 23.4% (n=33) were compliant with app usage for 25% or less of the study period, and 28.4% (n=40) of participants were compliant with app usage for 75% or more of the study period.

One-quarter of study participants attended multiple educational webinar group sessions (3 or more sessions attended). One-half of study participants did not attend any of the educational webinar group sessions.

The web-based week 12 survey was sent electronically to participants after completion of coaching session 6 and was returned by 78.7% (n=111) of participants. The web-based week 24 survey was sent electronically to participants after 24 weeks had elapsed since the start of the study; 73.9% (n=54) of participants in arm 1 and 51.5% (n=35) of participants in arm 2 completed the week 24 survey. The differences in survey completion rates between the 2 treatment arms may be attributable to the difference in level of engagement with Wellinks; specifically, it is possible that arm 2 participants who had less engagement with the Wellinks team between week 12 and week 24 had less interest or motivation in returning the survey.

#### Satisfaction Metrics

Participants were asked via survey at week 12 and week 24 to indicate their level of agreement with various statements aimed to understand whether they valued individual components of the Wellinks solution. Options included strongly agree, agree, neither agree nor disagree, disagree, and strongly disagree. Table 2 presents the proportion of participants who indicated they strongly agreed or agreed with each statement at the end of the study (week 24). Data are shown for the combined cohort not separated by treatment arm due to similar findings across the arms. Only 1 statement appeared to reflect a difference by treatment arm at the end of the study: 92.6% (n=50) of respondents in arm 1 and 68.6% (n=24) of respondents in arm 2 strongly agreed or agreed that “using the Wellinks solution has helped me to learn more about my COPD.” This difference may reflect the higher engagement with health coaches in arm 1 throughout the second half of the study period. There was a low level of disagreement with any of these statements indicating that most study participants find value in the components of the Wellinks solution and the entirety of the offering, regardless of group assignment.

**Table 2:** Participant agreement with statements about intervention components^a^.

Statement	Participants who agreed or strongly agreed, n (%)
I think having access to educational content is valuable.	85 (95)
Overall, I found the Wellinks solution to be valuable*.*	84 (94)
I think having meetings with my health coach is valuable.	81 (91)
I think being able to message my health coach is valuable.	81 (91)
I think being able to take and log pulse oximeter measurements at home is valuable.	80 (90)
I found the Wellinks app easy to use*.*	79 (89)
I think being able to take and log spirometry measurements at home is valuable.	75 (84)
Using the Wellinks solution has helped me to learn more about my COPD^b^.	74 (83)
Using the Wellinks solution has helped me to manage my COPD better.	72 (81)
I think being able to track and log my symptoms in the app is valuable.	66 (74)
I think having my medication schedule in the app is valuable.	55 (62)
Using the Wellinks solution has helped me to take my COPD medication as needed.	39 (44)

^a^Survey responses are from 89 participants who completed these questions in the end-of-study survey at week 24 (n=54 from arm 1 and n=35 from arm 2). The proportion of participants who selected that they “agreed” or “strongly agreed” with each statement is shown. Statements are listed in rank order from the statement with the highest degree of agreement to the lowest.

^b^COPD: chronic obstructive pulmonary disease.

#### Net Promoter Score

Participants were asked after week 12 and week 24 whether they would recommend Wellinks to friends, family members, or associates who also live with COPD to determine the NPS. Overall, the week 12 NPS was +57, and the week 24 NPS was +60. NPS differed by assigned treatment arm; NPS for arm 1 and arm 2 was +64 and +55, respectively, at week 24.

### Clinical Outcomes

#### COPD Self-Efficacy Scale

CSES scores were collected at baseline, week 12, and week 24 through a web-based survey. At baseline, the mean total score was 103.9 (SD 28.71), with the lowest domain scores on average observed for physical exertion and weather or environmental factors.

The CSES total score significantly improved from baseline to the end of the first 12-week study period during which all participants received the same level of coaching (LS mean change from baseline 11.1, SE 3.10; *P*<.001; n=96). These improvements were sustained across the entire study cohort at week 24 (LS mean change from baseline 23.6, SE 4.81; *P*<.001; n=77).

After week 12, participants were split by assignment to arm 1 or arm 2. Significant improvements in total CSES score from week 12 to week 24 were also observed in arm 1 (LS mean change 8.6, SE 4.04; *P*=.04; n=38) and arm 2 (LS mean change 10.6, SE 4.33; *P*=.02; n=34). In total, 5 participants did not complete CSES at week 12 but did complete the CSES at week 24.

Scores in all domains were significantly improved from baseline to end of the study in both arms (*P*<.001 for all domain comparisons except arm 2 for negative affect [*P*=.006] and intense emotional arousal [*P*=.002]). The minimally clinically important difference for CSES has not been found in the literature. The greatest differences were observed in the physical exertion and behavioral risk factors domain as shown in Table 3.

**Table 3:** COPD^a^ Self-Efficacy Scale domain scores.

Domain	Week 12 (n=96)			Week 24 (n=77)		
	Score^b^	LS^c^ mean change (SE)	*P* value	Score^b^	LS^c^ mean change (SE)	*P* value
Negative affect	3.6	0.3 (0.10)	.01	3.8	0.6 (0.15)	<.001
Intense emotional arousal	3.6	0.2 (0.09)	.009	3.9	0.6 (0.14)	<.001
Physical exertion	3.1	0.5 (0.11)	<.001	3.5	0.9 (0.17)	<.001
Weather or environmental factors	3.2	0.4 (0.10)	<.001	3.6	0.8 (0.15)	<.001
Behavioral risk factors	3.4	0.4 (0.11)	<.001	3.7	0.8 (0.16)	<.001

^a^COPD: chronic obstructive pulmonary disease.

^b^Domain scores have a scale of 1-5, calculated as the mean rating of each domain question.

^c^LS: least squares.

#### mMRC Dyspnea Scale

The baseline mMRC scores reflect variability in the study population regarding dyspnea. mMRC scores range from 0 (“I get out of breath only when I engage in strenuous exercise”) to 4 (“I am often too out of breath to leave the house, or I get out of breath even when dressing”). The population mean score was 2.0 (SD 1.26) at baseline. The distribution of baseline scores and the mMRC response rates at week 12 and week 24 can be observed in Table 4. In total, 31.6% (n=30) of participants improved by at least 1 category on mMRC from baseline to week 12, and 35.1% (n=27) improved from baseline to week 24. No differences between treatment arms were observed from week 12 to week 24 (*P*=.77, chi-square test).

**Table 4:** mMRC^a^ responder analysis. Baseline mMRC scores are shown for the study population (n=141).

Parameter		Values, n (%)
**mMRC score at baseline (n=141)**		
	0: I get out of breath only when I engage in strenuous exercise.	13 (9.2)
	1: I get out of breath when I am hurrying or walking up a slight hill.	47 (33.3)
	2: I walk slower than others of my age because I am out of breath, or I have to stop often to catch my breath.	38 (26.9)
	3: I have to stop for breath after walking for ~100 yards.	16 (11.3)
	4: I am often too out of breath to leave the house, or I get out of breath even when I am getting dressed.	27 (19.1)
**Week 12 responder status^b^ (n=95)**		
	Improved^c^	30 (31.6)
	No change	53 (55.8)
	Worsened^d^	12 (12.6)
**Week 24 (end of study) responder status^e^ (n=77)**		
	Improved^c^	27 (35.1)
	No change	36 (46.8)
	Worsened^d^	14 (18.2)

^a^mMRC: Modified Medical Research Council.

^b^Week 12 responder status is reported for 95 participants for whom mMRC data were available at baseline and week 12.

^c^Participants were indicated to have “improved” if their score decreased by one or more points from baseline.

^d^Participants were indicated to have “worsened” if their score increased by one or more points from baseline.

^e^Week 24 responder status is reported for 77 participants for whom mMRC data were available at baseline and week 24.

#### Pulmonary Function and Pulse Oximetry

Interpretation of the FEV_1_, PEF, and SpO_2_ data collected in this study was limited by the small sample size and the declining use of the connected devices throughout the study period. Use of the pulse oximeter waned over the study period: 126 (89.4%) were compliant with pulse oximeter use in study week 1, which fell to 60 (42.6%) in study week 12, and further to 13 (9.2%) in study week 24. Use of the spirometer also waned over the study period: 116 (82.3%) were compliant with spirometer use in study week 1, which fell to 59 (41.8%) at study week 12, and further to 12 (8.5%) at study week 24. Based on the limited data set, these data cannot be reliably analyzed to make any conclusions about changes in pulmonary function or pulse oximetry throughout the study.

#### Patient-Reported HRU

Interpretation of the patient-reported HRU data is limited by the infrequency of events reported. In total, 90% (n=127) and 93.6% (n=132) of study participants reported no COPD-related emergency room visits or hospitalizations, respectively, in the 3 months prior to baseline. Similarly, 89.6% (n=95) and 93.4% (n=99) of study participants reported no COPD-related emergency room visits or hospitalizations, respectively, during the study period. As expected, physician visits were more common than emergency room visits or hospitalizations. However, at all time points, participants most commonly reported none or only 1 COPD-related physician visit for the prior 3-month period. The event rate for physician visits is thus also inadequate for detection of any impact of Wellinks; furthermore, there is inadequate data to analyze any effect by treatment group.

### Safety

No adverse events were reported by the participants during the study period.

## Discussion

### Principal Results

Since the Wellinks clinical model has evolved over time to include the availability of respiratory therapy and health coaching services, questions emerged as to whether the target population—people living with COPD—would engage as they had done in a previously reported pilot study of a more basic program offering [8] and whether data may be collected to better optimize the frequency and duration of the personal health coaching component. Thus, the purpose of this 2-arm study design was to collect clinical and nonclinical data to optimize the appropriate duration and frequency of health coaching with Wellinks.

Study participants displayed a high degree of engagement with the health coaching component of Wellinks. By contrast, the study population had substantial attrition in the use of the mobile app and connected devices throughout the entire study duration despite rating all of them as highly valuable.

Importantly, this study provided evidence for the first time of the clinical value of patient participation in Wellinks. Significant improvements in COPD self-efficacy and breathlessness (mMRC) were observed for study participants regardless of assignment to arm 1 or arm 2. The improvement in the CSES observed after engaging in the Wellinks program may reflect the increased belief among study participants in their own ability to do certain activities, despite any potential perceived limitations due to their COPD diagnosis. Improved confidence in physical activities would be predicted to perpetuate a greater level of physical activity, and associated health outcome improvements may result.

Approximately one-third of participants demonstrated an improvement in breathlessness over the course of the study; improvements in shortness of breath as measured by mMRC are also reflected in the improvements in CSES, wherein the greatest degree of improvement was observed in the physical exertion domain. Although this study design does not allow for clear causal relationships to be determined, one hypothesis is that the remote pulmonary rehabilitation and education provided by the Wellinks health coaches may have improved breathlessness, which then also resulted in greater confidence (self-efficacy) on the physical exertion domain of the CSES [9]. Taken together, we can infer that the use of Wellinks improved self-efficacy and breathlessness, which may predict an improvement in the quality of life for these patients.

One difference between the treatment arms was observed in the NPS values. It is hypothesized that the higher NPS value observed for arm 1 compared to arm 2 may be attributable to the higher degree of interaction between health coaches and the study participants assigned to that arm. It has been frequently reported that digital interventions have the greatest value when combined with personal coaching or counseling [2,11,12], and this greater value may be reflected in the NPS.

### Limitations

The key limitation of this study is missing data for certain outcomes of interest, such as pulmonary function and HRU. Pulmonary function was assessed by way of home use of a Bluetooth-connected spirometer and pulse oximeter. Interpretation of these results is significantly limited by the lack of consistent use of these devices throughout the study period. Very low compliance with the study-directed use of once per week for each device resulted in a very small sample size, from which clear conclusions cannot be drawn. Although data interpretation is thus limited, this design was intentional to best reflect the real-world use of Wellinks; specifically, health coaches did not mandate the use of the devices but did remind participants to use them as appropriate.

Low long-term compliance with the connected devices is not an entirely surprising finding. It has been previously reported that remote monitoring alone with various biometric devices is subject to failure if it is not effectively integrated into the existing health care delivery model [3]. These results suggest that more needs to be done within the Wellinks clinical model to integrate the device data with the remote pulmonary rehabilitation and health coaching components of the program. It would be important to better understand whether the limited use of the devices was due to technical challenges, due to a lack of perceived value, or some other reason. If participants do not recognize the value of the data collected by these devices, it is possible that more can be done to educate patients about the information, provide context for interpreting the results that are recorded, and integrate health care providers into the process. Furthermore, the Wellinks model includes various components that allow for flexibility to meet patient needs; therefore, compliance with certain components may be expected to vary from individual to individual, in part as a reflection of different needs and preferences of each participating person.

HRU was assessed in this study based on participant self-report using a 3-month look-back period. The main limitations to interpreting these data are the low frequency of events reported and the recall bias that can result from this approach. Future studies of Wellinks will rely on verifiable information from electronic medical records or claims databases to inquire about HRU. Missing data were also the result of failure of some participants to complete all surveys per the study protocol. Notably, based on a meta-analysis of 1071 web-based surveys, completion rates average 44% [13], making this survey response rate better than average, although still an important limitation.

Additionally, the enrolled population may reflect a highly motivated subset of people living with COPD, given their existing engagement with the COPD Foundation prior to the study start; those who were recruited from the COPD Foundation Patient-Powered Research Network, by definition, have self-selected to contribute to research activities, which reflects a high degree of motivation. The same attributes may not be present in the general COPD population. Furthermore, the demographics of this study cohort may not fully reflect the demographics of the COPD population in the community. There was slightly more representation of females compared to males, which is consistent with observed trends of increased prevalence of COPD among women, while a decrease in prevalence has been observed in males over recent decades [14]. However, race and ethnicity are known to impact COPD risk but yet are not well represented in this study cohort [15].

### Comparison With Prior Work

It has been recognized in prior research that engagement with technology among older adults is dependent upon personal support from professionals or peers [16]. The high receptivity of this study population to personal health coaching sessions as compared to the low receptivity to the use of connected devices may also reflect this need for personal connection and support. In designing digital solutions for this population, it will be important to consider the value of the personal connection between the individual and their coach as a means to achieving greater adoption of associated technologies, such as the app and connected devices included in this study.

There are limitations to comparing the previously published pilot study of Wellinks to the study reported here; the populations differ in important ways (ie, in the pilot study, participants were recruited from a single provider’s practice, whereas in the ASPIRE study reported here, participants were recruited nonpersonally through the COPD Foundation), and the intervention differs as well (eg, the pilot study included most of the same technological components but lacked the health coaching component included here).

### Conclusions

This study demonstrates the interest and satisfaction of an ambulatory COPD population with the additional support and services provided by Wellinks. Health coaching appeared to be the most valuable component of Wellinks in this study. Signals of clinical outcome improvements in this study are encouraging and would best be further explored in larger cohorts to assess meaningful impact on a population level in terms of clinical improvement and impact on HRU.

Strategies and services to improve chronic disease self-management, such as with what Wellinks offers to patients with COPD, have been shown to reduce the burden of chronic diseases on individuals and the health care system at large. The data reported here are valuable not only to further optimize Wellinks but also to inform novel program design by others.

### Future Directions

Future studies will explore the ability of newer and modified versions of Wellinks to reduce hospital readmissions following an acute exacerbation as well as to explore the integration of Wellinks into a large accountable care organization. There remains a significant opportunity to bring remote disease management tools to people living with COPD, and these studies will further build the evidence base and support the long-term scalability of the program.

## Abbreviations

COPD: chronic obstructive pulmonary disease
CSES: COPD Self-Efficacy Scale
FEV_1_: forced expiratory volume in 1 second
HRU: healthcare resource utilization
IRB: institutional review board
LS: least squares
mMRC: Modified Medical Research Council
NPS: net promoter score
PEF: peak expiratory flow
SpO_2_: saturation of peripheral oxygen

## Data Availability

The data sets generated during this study are available from the corresponding author on reasonable request.
